# A rare case of membranous nephropathy associated with chronic inflammatory demyelinating polyradiculoneuropathy

**DOI:** 10.1080/0886022X.2023.2209659

**Published:** 2023-06-05

**Authors:** Qiong Zhang, Ying Tan, Lingchao Meng, Rong Xu, Fude Zhou, Minghui Zhao

**Affiliations:** a Renal Division, Department of Medicine, Peking University First Hospital, Beijing, China; b Institute of Nephrology, Peking University, Beijing, China; c Key Laboratory of Renal Disease, Ministry of Health of China, Beijing, China; d Key Laboratory of CKD Prevention and Treatment, Ministry of Education of China, Beijing, China; e Research Units of Diagnosis and Treatment of Immune-mediated Kidney Diseases, Chinese Academy of Medical Sciences, Beijing, China; f Department of Nephrology, The Fifth Hospital of Shanxi Medical University, Jinzhong, China; g Department of Neurology, Peking University First Hospital, Beijing, China; h Peking-Tsinghua Center for Life Sciences, Beijing, China

Membranous nephropathy (MN) is a major cause of nephrotic syndrome in adults. Some cases of atypical MN have been reported concomitant with chronic inflammatory demyelinating polyradiculoneuropathy (CIDP) [1–3]. However, the relationship and mechanism remain unclear. Antibodies against different subtypes of neurofascin and contactin have been detected in a small number of CIDP patients [4,5]. Cases of concurrent CIDP and nephrotic syndrome have been reported and indicated that neuro-renal disease might be mediated by autoantibodies targeting myelin and podocytes. Twenty-seven cases of CIDP with MN were identified in literature, among which four patients had CIDP and MN symptoms at the same time, 16 patients had CIDP symptoms before MN, and two patients had MN clinical manifestations before CIDP nervous system symptoms. Five patients were reported positive for circulating anti-contactin 1 antibodies (anti-CNTN1) [1–4].

Here, we report the case of a patient who developed atypical MN after the onset of CIDP. The coincidence of the onset time and the good response of MN and CIDP after the application of biological agents offers a unifying hypothesis for neuro-renal diseases. However, none of the known CIDP-related antibodies, such as anti-NF-155, anti-NF-186, anti-CNTN1, anti-CNTN2, or anti-CASPR1, were discovered in our cases. The negative results of the currently available autoantibodies were different from those of previous reports, which indicated that there might be other potential common antigens and a common immunopathogenesis in the association of MN and CIDP deserves further scrutiny.

A 67-year-old Chinese man presented with a 9-month history of progressive numbness in all four extremities and a 4-month history of lower extremity edema. Two months prior, the numbness of the limbs had increased gradually with weakness and severe edema, and the patient was unable to walk. On evaluation at another hospital, initial laboratory studies revealed a serum creatinine 149.4 µmol/L (44–133 µmol/L) (corresponding to an estimated glomerular filtration rate of 41.18 mL/min/1.73 Å m^2^ as calculated by the CKD-EPI equation), serum albumin 22.7 g/L, and proteinuria 26 g/day (total volume 1400 mL). Cerebrospinal fluid (CSF) examination showed protein 118–187 mg/dL, white blood cell count 34/mm^3^, and Pandy’s test was positive. CSF anti-sulfatide IgG antibody was positive. Sural nerve biopsy showed mildly reduced myelinated nerve fiber density and thinly myelinated fibers, with CD 68 positive macrophages infiltration in the epineurium and endoneurium, which corresponded to CIDP (Supplementary Item S1). Cranial MRI, contrast-enhanced MRI of the cervical and thoracic spine revealed no abnormal enhancement of the spinal canal. Therefore, the patient was diagnosed with CIDP. The patient was treated with methylprednisolone (80 mg/day for five days) and intravenous immunoglobulin (27.5 g/d for five days, 30 g/d for five days) simultaneously at another hospital.

After the initial treatment, he reported a decrease in the numbness of four extremities and was able to move slowly. Upon admission, sensory examination revealed decreased sensation to pinprick extending to the wrists in the arms and to the ankles in the legs. The decreased vibration sensation of lower limbs was also observed. Romberg’s test was positive, suggesting the sensory ataxia. Deep tendon reflexes were absent in the four extremities bilaterally. In addition, mild edema of both lower limbs was observed. The remainder of the examination was normal. The modified Rankin Scale (mRS) score was 3. Serum anti-M-type phospholipase A2 receptor (PLA2R) antibody was measured by enzyme-linked sorbent assay. Cell-based assays for anti-neurofascin-155 (NF-155), anti-NF-186, anti-contactin-1 (CNTN1), anti-CNTN2, and anti-contactin-associated protein-1 (Caspr1) antibodies reactivity were performed as previously described in literature [4]. The results were all negative. The motor nerve conduction study showed abnormal results, mainly suggesting the demyelinating features (Supplementary Item S1). To clarify the pathological changes of his kidney histology, a renal biopsy was performed (Figure 1). Direct immunofluorescence showed staining along capillary loops (IgG (3+), IgM (1+), C3 (2+), kappa (3+), lambda (3+), IgG1 (2+), and IgG2 (2+)). Light microscopic examination showed that 3/22 glomeruli were ischemic and sclerotic and 2/22 were glomerulosclerosis. The rest of the glomeruli showed mild proliferation of mesangial cells and stroma. The glomerular basement membrane thickened diffusely, with the formation of segmental spikes. Immunohistochemistry showed PLA2R negative. Electron microscopy revealed uniform diffuse thickening of the glomerular basement membrane with the formation of segmental spikes, abundant subepithelial electron-dense deposits and diffuse foot process effacement consistent with atypical MN with glomerular hypertrophy and ischemic renal injury.

**Figure 1.**
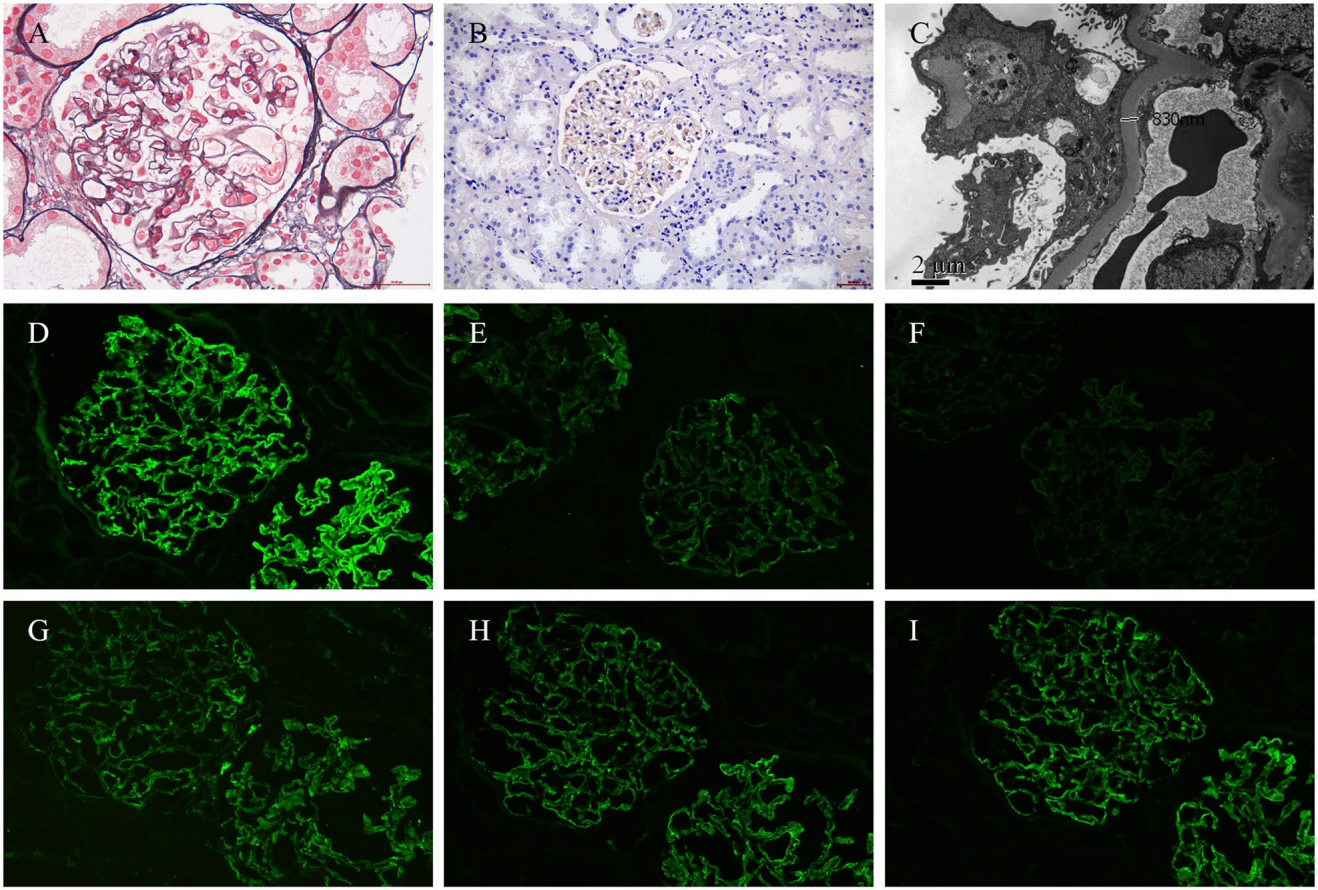
Histologic findings on renal biopsy. (A) Light microscopy showed the glomerular basement membrane thickened diffusely, with the formation of segmental spikes (periodic methenamine silver and Masson trichrome staining, ×400); (B) immunohistochemical staining of phospholipase A2 receptor (×200); (C) electron microscopy showed uniform diffuse thickening of the glomerular basement membrane (830 nm) with the formation of segmental spikes; (D–I) immunofluorescent staining for IgG, IgG1, IgG2, C3, kappa, and lambda granular staining along glomerular basement membrane.

The patient was administered intravenous methylprednisolone 40 mg daily for approximately two weeks, followed by oral prednisone, and plasma exchange was performed five times. Neurological symptoms such as ataxia, limb numbness, and walking gait were slightly alleviated, the mRS score was 2, and the nephrotic syndrome was slightly improved. The patient had massive proteinuria and elevated serum creatinine levels. After 2–4 weeks of rituximab treatment, neurological symptoms gradually disappeared, the serum creatinine was gradually reduced from 170.7 µmol/L to 102 µmol/L. The urinary protein was reduced to <0.15 g/24 h in 13 months. The patient remained stable for the last 22 months during follow-up, with no relapse of neurological symptoms (Figure 2).

**Figure 2.**
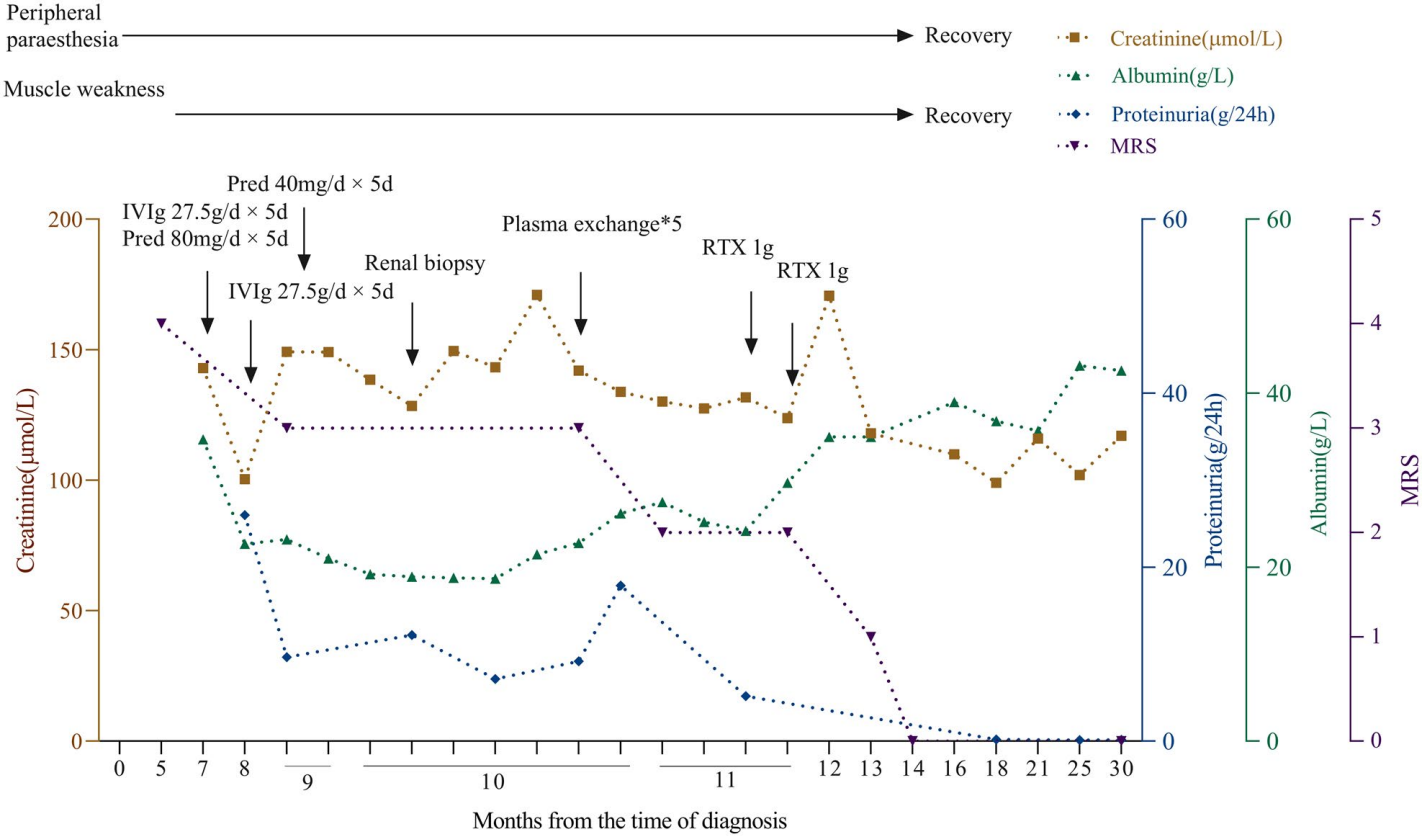
The clinical course of the current case. RTX: rituximab; IVIg: intravenous immunoglobulin; Pred: prednisone; MRS: modified Rankin Scale.

## Discussion

MN can be classified into idiopathic membranous nephropathy (iMN) and secondary membranous nephropathy (sMN). The pathological features of iMN and sMN are similar in glomerular basement membrane lesions, but mesangial or endothelial cell proliferation and multiple locations of immune complex deposition could be found only in sMN, mostly without PLA2R and predominant IgG4 deposition. Our case showed negative renal staining of IgG4 and PLA2R, and mild proliferation of mesangial cells and stroma. Thus, our case was more likely to be sMN.

Renal manifestations of our case occurred five months after the onset of CIDP. Our case presented with progressive symmetric, distal muscle weakness of upper and lower limbs, and sensory involvement in the four extremities bilaterally, developing over 8 weeks, reduced tendon reflexes in all limbs. The nerve conduction study proved demyelinating features and the CSF examination indicated albumin-cytologic dissociation. The sural biopsy showed thinly myelinated fibers with macrophages infiltration. For the patient, the immunosuppression treatment was effective, and no other cause of neuropathy was found. According to the EAN/PNS diagnostic criteria, the CIDP should be considered for our case [6].

Cases of concurrent CIDP and nephrotic syndrome have been reported, indicating that neuro-renal disease may be mediated by autoantibodies targeting myelin and podocytes [7,8]. The presence of autoantibodies is thought to be responsible for special clinical features and associations as well as treatment responses. In the current case report of CIDP with MN, the optimal treatment for these cases remains unknown. Most patients with CIDP and concomitant MN (15/21, 71%) initially responded well to glucocorticoid and immunotherapies. By literature survey, and a more favorable response to immunotherapies compared with anti-CNTN1 antibody-positive CIDP [3].

Unlike other reported cases, our patient did not seem to respond well to glucocorticoid or intravenous immunoglobulin. The clinical symptoms of CIDP and proteinuria level of MN were gradually relieved after plasma exchange and rituximab nearly simultaneous, suggesting an autoimmune etiology of CIDP and MN. The combination of CIDP and nephrotic syndrome may share the same autoimmune etiology. Rituximab can be used for refractory cases.

## Supplementary Material

Supplemental Material
