# Supplementary material for: A rare case of membranous nephropathy associated with chronic inflammatory demyelinating polyradiculoneuropathy
Source: Ren Fail. 2023 Jun 5;45(1):2209659. doi: 10.1080/0886022X.2023.2209659 (PMC10243384; doi:10.1080/0886022X.2023.2209659)
Supplement: Supplemental Material [file IRNF_A_2209659_SM6094.pdf]

Figure legend

Figure S1 Histologic findings on sural nerve biopsy. (A) The HE staining indicated no vasculitis or abnormal material deposition in the epineurium, perineurium and endoneurium (×100). (B) The immunohistochemical staining for CD 68 showed small amount of macrophages infiltration in the epineurium and endoneurium (×200), but (C) no CD8 positive lymphocytes infiltration (×200). (D) The semithin section of toluidine blue staining showed mildly reduced myelinated nerve fiber density and the thinly myelinated fiber (arrow) (×400).

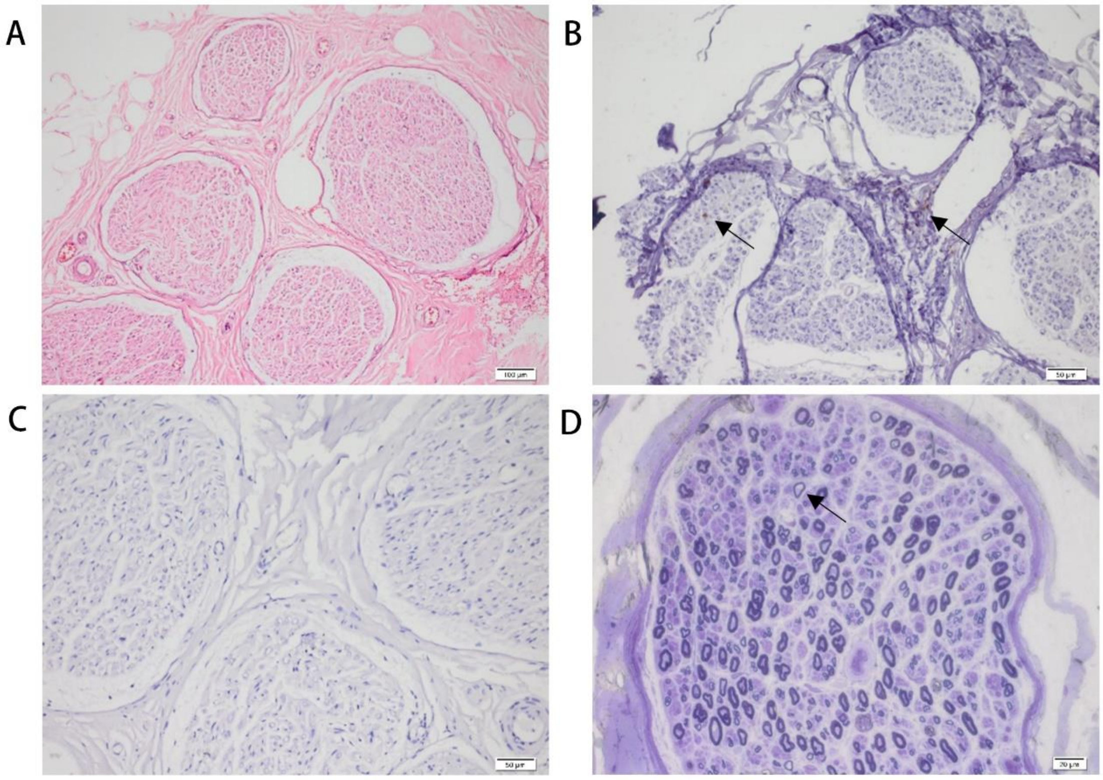

Information Classification: General

Table S1. The result of nerve conduction study of the patient

	Latency(ms)	Amplitude(mV)	NCV(m/s)
**Motor NCS**			
**L Median nerve**			
Wrist	4.13	7.0	
Elbow	9.13	6.9	50.0
**R Median nerve**			
Wrist	4.52	6.4	
Elbow	9.3	5.1	46.3
**L Ulnar nerve**			
Wrist	3.7	6.0	
Below elbow	7.35	5.1	46.6
**R Ulnar nerve**			
Wrist	4.08	6.7	
Below elbow	7.73	6.8	46.6
**L Peroneal nerve**			
Ankle	8.03	0.93	
Below fibular head	18.5	0.60	26.3
**R Peroneal nerve**			
Ankle	8.03	0.63	
Below fibular head	17.3	0.44	30.7
**L Tibial nerve**			
Ankle	10.7	2.7	
Popliteal fossa	20.2	1.16	38.9
**R Peroneal nerve**			
Ankle	9.12	1.69	
Popliteal fossa	19.2	1.67	36.7
**Sensory NCS**			
Median nerve	NR	NR	NR
Ulnar nerve	NR	NR	NR
Radial nerve	NR	NR	NR
Sural nerve	NR	NR	NR
Superficial peroneal nerve	NR	NR	NR

Information Classification: General
